# Developing Geographical Narratives: Pupils Create Digital Text Adventures with Twine

**DOI:** 10.3390/ejihpe10040078

**Published:** 2020-12-03

**Authors:** Veit Maier, Alexandra Budke

**Affiliations:** Institute of Geography Education, University of Cologne, Gronewaldstraße 2, 50931 Cologne, Germany; alexandra.budke@uni-koeln.de

**Keywords:** text adventure, Twine, narrative, creativity, collaborative creativity, computer games, applying geographical knowledge, geography education

## Abstract

Applying geographical knowledge in new contexts is a creative and difficult task for school pupils. However, creating text adventures with the open-source tool Twine may be one way to apply geographic knowledge, but there is currently no research that confirms this. We attempted to determine how pupils in small groups constructed text adventures in geography lessons, focused on the topic “Tourism in Myanmar: threat or opportunity”. We recorded the construction processes of 14 pupils audibly, organized into six teams, and analyzed their games. We found that the different text adventure construction activities between the groups had minimal differences. The groups predominantly asked questions and expressed ideas that used meta-conversation for organization and used agreements. These and other text adventure construction activities can help to specify a model of collaborative creativity. In addition, successful groups wrote geographical narratives with adverbs to emphasize the psychological proximity, rhetorical questions and feelings in their stories, and used more words than the others. The results suggest a focus of future research should be on developing a model for integrating geographical narrative skills into geography lessons and intensifying research about collaborative creativity.

## 1. Digital Media as an Opportunity

Computer games are a popular leisure activity for student-aged children. Around 73% of all young people (12–19 years) play computer games at least once a fortnight [1] (p. 55). Many of these computer games deal with geographical issues such as (sustainable) resource use, migration, urban development, and tourism (e.g., Age of Empires, Anno 1800 and Civilization VI [2] (pp. 10–14)). These issues are relevant to school geography curriculum topics, and as students enjoy these topics in games, educational institutes should pay attention to this in formal education, too, including how commercial games could be integrated into the classroom. Previous studies on the use of digital games in school lessons have shown many positive effects on learning, with these games motivating students in a variety of ways [3,4]. Players can try out different roles within the game, which is not possible in the real world. The games also provide faster feedback on decisions than in real life [4]. Herbert et al. also found a development of geographic learning knowledge in pupils from interacting with geographical serious games [5].

Using digital games in lessons could be a chance to renew methodical toolboxes and teaching ideas and motivate pupils with creative tasks. However, little is known about the frequency of use of digital games in class; a study in 2018 found that only 24% of teachers in Germany used digital media in teaching daily [6] (p. 215), which could explain why only 9% of the pupils in North Rhine-Westphalia (Germany) use information and communication technology for school-related purposes outside of school [7] (p. 120) (the lowest percentage in an international comparison). The COVID-19 pandemic has meant that this situation is currently changing, with many lessons now taught online. At present, we can only assume that some of the changes in media use, methods or attitudes will continue to be used.

In the context of the low integration of digital media in formal education and the reported opportunities this could offer, creating digital games could also be an approach to stimulating students’ engagement with content issues and initiate deeper learning processes. One piece of software that could be used for this purpose is “Twine” (Twinery.org). Twine is “an open-source tool for telling interactive nonlinear stories” [8], and it can be used to create text adventures. Text adventures are text-based games, and developing these with Twine is a creative writing process [9] (pp. 137ff). Historically, travelogues are thought to be the first geographical information with which people collected information about unknown countries. Today, narratives are (again) gaining importance in social science because narrative discourses play a decisive role in the construction of reality (cf. the linguistic turn, [10]). In geography lessons, students learn with and about text, as words construct our world and language. On this basis, it could be a great opportunity, especially in geography education, to access the world with narratives written in Twine. However, there is no previous research that deals with the use of Twine in the context of geography lessons or geographical topics. Therefore, we do not know how the creative writing process works within geographical contexts and how far this method develops learning outcomes. Our research questions are the following:

### How Do Pupils in Small Groups Construct Creative Text Adventures on Geographical Issues?

The following questions are subordinate to the research question:

1. How do the participating pupils evaluate working with Twine?
2. How does the creative process work when constructing text adventures with Twine on geographical issues?
3. What are the differences between the groups that construct creative text adventures with Twine on geographical issues?

We analyzed a text adventure construction process, which took place in groups, and the text adventure games produced by a set of 11th grade students, by examining the conversation and the games in a qualitative content analysis. All the games were constructed in geography lessons with the topic “Tourism in Myanmar: threat or opportunity”. We chose this topic because the impact of tourism is a typical topic in geography lessons, and because different stakeholders, such as tourists, the tourist industry, politicians, etc., have diverse narrative possibilities associated with them, which could be represented in the game. The article begins with a theoretical overview, going on to explain the methodology. The results are presented according to the research questions, and a discussion and conclusion with the limitations and implications of the results follow.

## 2. Theoretical Background

For a broader understanding of this article, we begin this section with an overview of digital media in geography lessons. The following section examines the text form narratives in geography before we look more closely at Twine and digital games based on narratives. As constructing a game can be described as problem solving [11] (pp. 217ff), we present relevant information about problem solving and about the creativity that follows.

### 2.1. Digital Media in Geography Lessons

The starting point for the study of digital media and geography education in Germany was the implementation of GIS (Geoinformation Systems) in geography teaching in the early 1990s [12] (p. 69). Subsequently, didactic applications for further “new” media in teaching were developed; the significance of e-learning for global learning was discussed [13]; flipped classroom modules for geography teacher training were presented, e.g., [14]; and new digital teaching media for geography teaching were developed and evaluated, such as virtual excursions, e.g., [15]. 

However, a deficit can still be observed: In a study on the use of geomedia, around a third of the interviewed pupils said that they never used the Internet or PCs in geography lessons [16] (pp. 149ff). The International Computer and Information Literacy Study (ICILS) 2013 found that only 1.6% of the pupils worked with a computer daily in school [17] (p. 121). This deficit is partly attributed to didactic concepts and examples of the meaningful integration of digital media into teaching not yet being sufficiently available or systematically implemented. As the demand to implement digital media in schools is great, the government in North Rhine-Westphalia invested around EUR 7 billion in the digitization of schools between 2017 and 2020 [18]. With the beginning of the COVID-19 pandemic, the situation changed suddenly from digital catch-up to a digital tight spot. As the pandemic continues, there can be no final conclusions drawn yet on the consequences for the education sector.

Constructing text adventures is particularly challenging, as geographical content that is digitally represented must be not only received but also created. Various studies have shown that programming, designing and creating computer games in educational contexts offer advantages over simply playing or receiving computer games. Kafai [19] (p. 39) discussed greater learning advantages or deeper reflection that goes beyond traditional learning when constructing computer games. Ke [20] recognized a deeper reflection on everyday mathematical experiences in children. Khalili et al. [21] identified greater accuracy in scientific facts and greater self-responsibility in children. 

Studies comparing playing with the construction of computer games found that “constructing a game might be a better way to enhance student motivation and deep learning than playing an existing game” [22] (p. 127), although the analyzed game in the quoted study was a memory game and no complex decisions had to be made. Tran [23] investigated the game development of children with the game engine Twine. However, only a few pupils were examined, and no geographical topics were chosen. Tran [23] emphasizes linguistic competence development at the sentence level (e.g., if–then constructions). On the textual level, Twine enables the programming of complex adventure games. The formulation of if–then constructions makes it possible to clarify causal relationships such as cause–effect relationships. These are crucial, for example, when constructing an Ishikawa diagram on various geographical topics. The creation of text coherence allows complex issues to be presented in a coherent way. This is important when arguing and justifying, and especially important when dealing with complex realities in geography such as migration, climate change or tourism. In our study, *justification* was a category for analyzing group conversation.

For telling interactive stories with Twine, there is no coding necessary and the tool can be used online at http://www.twinery.org/. Currently, no study has been undertaken that focuses on the use of Twine in geography education. We created the term “text adventure construction activity” to identify typical activities in the process of writing a text adventure. These activities are interesting in respect of comparing the construction processes for different text adventures. 

Traditionally, pupils create much inert knowledge that is not useable in lessons [24] (p. 102). To make this knowledge applicable, it has to be learned and applied in a contextualized way to generate established knowledge [25,26,27]. Creating text adventure games in geography lessons with geographical content is a learning opportunity that involves applying this knowledge to new areas. For example, knowledge about different travel behavior (from mass tourism to sustainable tourism) is difficult to apply in school. By creating text adventures, pupils are given the opportunity to create decision-making situations and anticipate their consequences. For example, in the case of tourism, the consequences of mass tourism and soft tourism could be presented in a text adventure game. The player would have to choose a form of tourism in the game and would thus experience the consequences. In these lessons, pupils must analyze, reflect on and reconstruct the available options in a digital environment, the systemic relationships and the argumentative justifications for decisions on spatial issues. Therefore, the development of interesting narratives is important, which is the central theme of the game’s plot and motivates engagement with the game.

### 2.2. Narrative and Geography

Narration plays an important role in many digital games. A narratologist in game studies understand games as stories and tries to analyze them with text-analyzing instruments. Bhatty [28] considered computer games as narrations with different narrative degrees of freedom for the player. On the other hand, ludologists understand computer games as games with rules and gameplay, and the way players interact with the game. Juul (2001) postulated that “you cannot have narration and interaction at the same time” and rejected the notion that players can be authors at the same time as controlling actions [29]. As text adventures can also be read as “interactive fiction” [30], we follow the narratologistic view and understand computer games as narratives. 

Narratives are as old as humankind. They are “*international, transhistorique, transculturel, le récit est là, comme la vie*” [31] (p. 102). Here, Barthes focuses on narratives as the mediation of sequences of events in different groups and societies in all time. We get to know and understand the social world through narrativity, as well as learning to give events meaning, and we constitute our social identities through narratives and narrativity [32] (p. 606). 

Narratives are traditionally analyzed in literature studies. Ehlich segregated narration into Narration 1 and Narration 2 [33] (p. 138f). Narration 1 is everyday narration such as reporting, describing, communicating etc. and is deemed to be narrating in a broader sense. It is described as the passing on of events. Meanwhile, Narration 2 is narrating in a narrower sense and describes the narrating of a story. Creating a common world is the defining characteristic of the two narration types, with stories having a specific structure: orientation is at the beginning, with a description of the place and the people; complication is in the middle—there is no story without it [34] (p. 141), and the resolution where the problem is solved is at the end. Furthermore, Boueke [35] identified some specific words, such as “suddenly”, and adverbs to emphasize psychological proximity in stories. In our study, we deal with narratives about geographical issues written by pupils who are trying to create a common world. Therefore, we consider geographical narrations to be Narration 2 (in a narrow sense) with described structural design with geographical content. 

Stories are considered to be 22 times easier to remember than simple facts [36], which is why many brands use storytelling methods to get us to remember and then buy products, and some mnemonic techniques (e.g., story or sentence mnemonics) use stories, too. Our brain remembers meaning better than literal wording [37]. Meaning can be transmitted better by stories than by telling simple facts because stories already connect facts in a network. Good stories are able to arouse emotions that help the audience to follow and to remember the story, too. Therefore, the development of geographical stories in the context of digital games may be a suitable approach to conveying geographical knowledge and understanding.

Recently, in geography and other social sciences, narratives and discourse analysis have been gaining importance. We call the pluralization of the disciplines researching narratives the “narrative turn”. In this context, narration is seen as a construction process that is examined in cultural and social sciences [38]. For example, Dickel (2006) focuses on travel narrations as mediations of conceptions of reality, criticizing German geography textbooks for not reflecting many authors’ texts accurately. “Thus, ‘objective experiences’ are conveyed in a supposedly subjective way” [39] (p. 26). This is particularly counterproductive with regard to a science propaedeutic approach. Dickel points out that during travel, (factual) knowledge can be compared with literature [39] (p. 39f). Narratives do not show events and instead create them by their discursive character [40] (p. 66). This is one way of using narrations regarding their real information content. In order to incorporate factual information into narratives, previous knowledge and research are essential. In our study, *research* was used as a category to analyze group conversations. 

In geography education, narrations play an important role in the consideration of nomothetical and ideographical approaches [41] (p. 71). On the one hand, abstract (natural) laws and facts are made more vividly understandable through narratives (e.g., by assigning small stories to stages of demographic transition), but on the other hand, personalized narratives from different stakeholders and perspectives are used to make different views clear, such as, for example, to make spatial conflicts and their social negotiation understandable [42,43]. The development of geographic narratives in digital games by students has not yet been empirically investigated, but it may be a creative and motivating approach to achieving deeper learning effects.

### 2.3. Problem Solving in Geography Education

Developing a text adventure game, for example, with Twine, is a game design process. Game design can be descried as complex problem solving [11], as the number of possible solutions is unknown and the problem is poorly defined. Furthermore, developing a text adventure game includes the design of decision situations. 

In the PISA report of 2012, the authors define problem solving as “an individual’s capacity to engage in cognitive processing to understand and resolve problem situations where a method of solution is not immediately obvious. It includes the willingness to engage with such situations in order to achieve one’s potential as a constructive and reflective citizen” [44] (p. 30). This definition includes the understanding of problems that “arise when a living being has a goal and does not know how to achieve that goal” [45] (p. 1). Furthermore, the definition is based on Dörner’s [46] (pp. 10ff) reference to “barriers”. Barriers include cognitive, motivational and affective factors [47]. This fits well with the individual writing of a Twine game.

In the PISA report 2015, the authors examine collaborative problem-solving competency and define it as “the capacity of an individual to effectively engage in a process whereby two or more agents attempt to solve a problem by sharing the understanding and effort required to come to a solution and pool their knowledge, skills and efforts to reach that solution.” [48] (p. 134). What is new is that, for the first time, collaborative problem solving is brought into focus. This fits well with the collaborative writing of a Twine game.

In 2012, the participating pupils from Germany scored above the OECD average but below the former group [44] (p. 53). In 2015, the participating pupils again performed above the OECD average. The fifteen year olds who attended a grammar school in Germany were particularly successful [49] (p. 70).

Individual problem-solving is an important skill in respect of an individualized society that the school law of NRW (Germany) also supports [50] (§1). Since our societies, and thereby geography education, too, are faced with many social challenges such as migration and global problems such as climate change, we should focus on collaborative problem-solving, too. In fact, the NRW School Act also demands social participation [50] (§2). The education standards in geography for the intermediate school certificate in Germany do not mention collaborative problem solving [51]. The standards for secondary education on a federal state level (NRW) mention “self-determined and community-related cooperation” [52] (p. 16). Collaborative problem solving is not mentioned at all. It therefore makes sense that, in a comparison of textbooks in the context of citizenship education, Maier and Budke [53] (p. 189) identified a focus on the individual in German textbooks and a focus on the community in textbooks from England and Wales.

It appears that, compared to the OECD average, German students are good problem solvers, albeit with a significant distance from the top group’s scores [44,49,54]. However, collaborative problem-solving competencies are not supported in the German curriculum for geography education. As geography deals with many global challenges, there seems to be a huge potential to learn and teach it in this subject. 

### 2.4. Creativity and Creative Writing in Geography

In the context of this study, the task of cooperatively designing a game focused on tourism in Myanmar in small groups was accompanied by three different challenges. The first challenge was creative cooperation between the text adventure authors. Social skills were required here to find the balance between creative freedom and goal orientation. The second challenge was developing several decision-making situations that defined a text adventure. This challenge must be understood as a problem of information processing [46] (p. 10). The initial state is known to us, but the target state, the means and the sequence are still unknown. The means are the decision-making situations, and creativity is needed to construct them. Thirdly, game design can also be understood as a problem-creating or problem-identification process, as every games needs a thematical conflict [28] (p. 132). The game designer must create or identify an inherent polytelic conflict or problem that gives the game its meaning [55] (p. 5). This identified problem must be understood as a problem of content or, in geography lessons, as a socially relevant problem such as climate change, migration or urban development [56] (p. 24). Creativity is needed to apply the general problem to the spatial example. Thus, pupils in school need to be creative in all understandings to undertake the task development process to produce a text adventure game with Twine. 

Creativity is generally characterized as combining the given materials in such a way that something new is created [57,58]. A *new idea* is the result and, in our study, a category for analyzing group conversation. Creative subjects are art and music. Nevertheless, geography contains some creative tasks, such as in the field of urban planning. For definition and some examples, see Maier and Budke [59] (p. 10). 

Creative writing is rarely used in science lessons. When pupils write, they summarize what they have learned in a summary sentence or they get dictated a text, for example [60] (p. 15). The same applies to geography education. One exception to this approach describes a study from Summetby-Murray. He experimented with using narrative inquiry as a creative writing technique in teaching geography students on undergraduate courses [61]. Narrative inquiry uses personal artefacts to understand the narrative of peoples’ lives [62]. He recognized the method’s suitability for fostering the connection between students’ life experiences and abstract concepts in cultural geography. Through narrative inquiry, students create their own reality in a reflective and constructivist manner [63]. This means that the construction of narratives and text adventures could be a creative way of applying geographical knowledge in geographical lessons.

**Figure 1:**
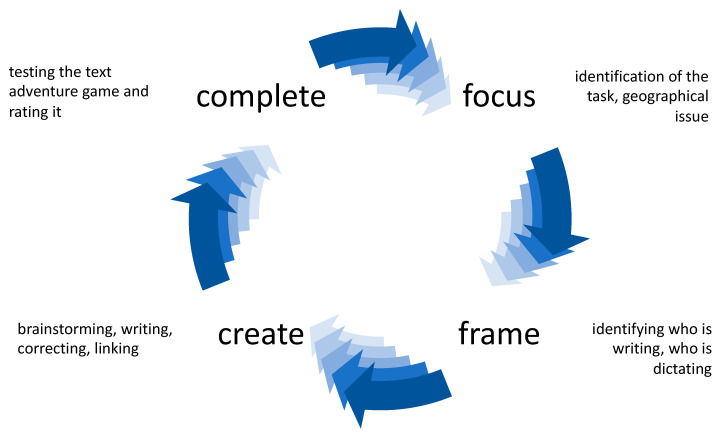
Four-stage model of collaborative creativity based on Aragon and Williams [66]. Own design.

The final report of PISA 2012 is titled “creative problem solving” [44]. However, Funke criticizes this title produced by the OECD and prefers the term “interactive” [64] (p. 100). He fears that readers might think that there is also uncreative problem solving. Wallas [65] describes four linear steps in individual creative problem solving: preparation in the sense of intensive thematic engagement, incubation as a maturing process, illumination as an insight phase, and the verification or systematic checking of solution ideas. In group creativity, often also described as collaborative or collective creativity, communication is the main factor for creative work [66]. In addition, the group and its members, the task and situational variables influence the cognitive, social and motivational team processes that promote group creativity [67] (p. 230). Aragon and Williams [68] developed a four-stage model of collaborative creativity (see Figure 1) on the basis of distributed groups (scientists and children). The Focus stage is the orientation task to identify the problem. The Frame stage describes the process of group organization. The next stage, Create, subsumes brainstorming, intense interaction between the group members and an intern evaluation process on the basis of arguments. The Complete stage is a test and external evaluation phase. All four stages are connected in an iterative process. As the model is intended to fit different collaborative creativity processes, it generalizes the process. Furthermore, it is not clear how the creative ideas arose in the collaborative process. Nevertheless, it offers the possibility to investigate collaborative creativity processes. If we want to adapt the general model to the text adventure game creation process, we need to consider the context of Twine and the topic “Tourism in Myanmar”. For a more detailed analysis, we wanted to derive text adventure construction activities. We understand text adventure construction activities as language actions [69] in the construction of text adventures. These are more specific in respect of the use of Twine, and they could help to elucidate how pupils in small groups construct creative text adventures. 

## 3. Materials and Methods 

This section is divided into information about the participants, the lesson, and the data collection and analysis. 

### 3.1. Participants 

We based this explorative study in a high school in the city of Cologne (Germany). The school is located in a quarter near the city center with a below-average number of migrants and below-average number of unemployed for the city [70] (p. 10). The observed class was a basic geography course. We observed 14 high school pupils (Gymnasium) (aged 17–18), organized into small teams, and how they constructed an adventure game with Twine on the topic of tourism in Myanmar. The participants or their parents gave their written consent for the recording, analysis and anonymized publication of the data. The pupils organized themselves into six small creation teams with two to three members. Almost half of the pupils (6 of 14) were male. None of the students had ever used Twine or heard of it before. We used small groups instead of individual game production to gain audio data of the discussions during the development process. A group size of two or three pupils is apparently relatively discursive [71].

### 3.2. Lessons on Tourism in Myanmar and Investigation 

**Figure 2:**
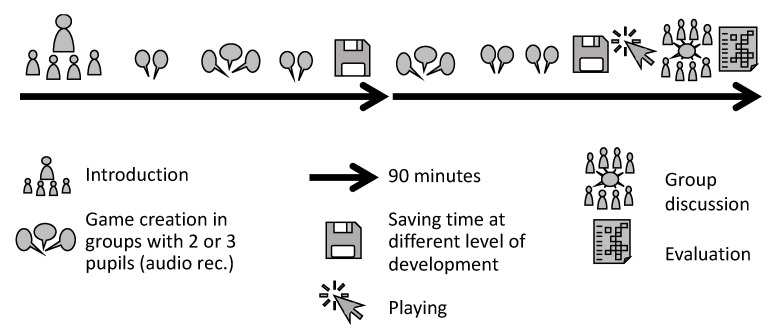
Procedure of the investigation.

To understand how the pupils constructed text adventures with Twine on the topic of “Tourism in Myanmar: threat or opportunity”, we taught two 90 min lessons in geography at school. In the lessons before the investigation (not in Figure 2), the teacher taught a unit about tourism in developing countries, using Thailand as an example. Tourism is embedded in the curriculum as “services in their importance for economic and employment structures” [72] (p. 19). The focus was on the impact of tourism as an economic factor in Thailand and the related land use conflicts. Understanding the causes, opportunities and risks is a prerequisite for reflecting on the possibilities for sustainable tourism development. In the final lesson before we started the study, the pupils were set a piece of homework to read a newspaper article about the political change and the development of tourism in Myanmar [73]. We choose the topic “Tourism in Myanmar: threat or opportunity” for these students as our topic because they would already have had previous and transferable knowledge on the subject. The political change in Myanmar and the development of tourism are an exciting topic because of their topicality, and they present a future-orientated question. The students received the following task: develop a text adventure with Twine on the topic “Tourism in Myanmar: threat or opportunity”.

The first 90 min of the study started with a 20 min introduction with explanations about text adventures, Twine and Myanmar (see Figure 2). The pupils were also given a scientific article about the development of tourism in Myanmar with a map and tables about tourist arrivals [74]. During the session, the pupils were able to use search engines on the Internet. Furthermore, we developed evaluation sheets together with some quality criteria for their adventures. Following this, the pupils started to create their first text adventures with Twine. The pupils worked on their games in the lessons only. In the second 90 min, the pupils continued creating their games. In the last 30 min, the pupils played their games and the games of the other groups and evaluated them with our evaluation sheet. The final task was a group discussion where we asked for the best game, with reasons, to gain qualitative criteria from the pupils as contextual material, in the case of difficulties in understanding the completed evaluation forms. The lesson ended with a questionnaire about working with Twine and text adventures, to enable an evaluation from the pupils’ points of view. 

### 3.3. Data Gaining and Data Analysis 

In line with the mixed-method approach, the qualitative data of the group conversations during the construction process were quantified in order to better compare the different groups and category distribution [75] (pp. 87ff). Furthermore, we gathered various data for triangulation (see Figure 2). We collected data through audio recordings of different text adventure construction processes in different small groups, games at two different level of development, and an evaluation questionnaire. As contextual material, we also recorded a plenary group discussion and evaluated the games’ ratings on jointly prepared evaluation questionnaires.

#### 3.3.1. Audio Recordings of Different Text Adventure Construction Processes

We recorded the groups’ construction processes. This is close to think aloud but without an artificial request to speak. Think aloud is effective in gaining insight into the thoughts and intentions of the person speaking [76]. We therefore did not gain an insight into inner speech [77] (p. 114), but we were able to identify how creating text adventures with Twine developed in the different groups. 

For the transcription of the audio files, we worked with transcription software (easytranscript). For the qualitative content analysis, we followed Mayring [78] and used MAXQDA. In iterative cycles, we inductive–deductively developed 9 categories or text adventure construction activities by summarizing, explicating and structuring [78] (p. 268f) the 1080 min of audio data in 1563 codes. These helped to answer questions about the construction process. We identified the following 9 text adventure construction activities (see Table 1). 

We summarized *ask questions and expressing ideas* because whether an idea is formulated into a sentence as a question or as a statement often depends on intonation only. Both intonations have a common reading in the material examined here: ideas are generated. The raising of the voice at the end of a sentence marks a question, and at the same time, it could express uncertainty or consideration. The uncertainty occurs because other ideas could be better, from consideration, and because ideas of the other group members are also welcome. 

The quantitative illustration of the qualitative data helped to interpret the qualitative data. This is particularly useful for identifying extreme cases, for example. The statements helped us to understand the content of the categories and to compare the different groups [75] (p. 87) [79]. For the analyses of the construction process for the Twine adventure games, we used an analysis of code relations. We included overlapping codes and codes that followed each other directly.

#### 3.3.2. The Games at Two Different Levels of Development

**Figure 3:**
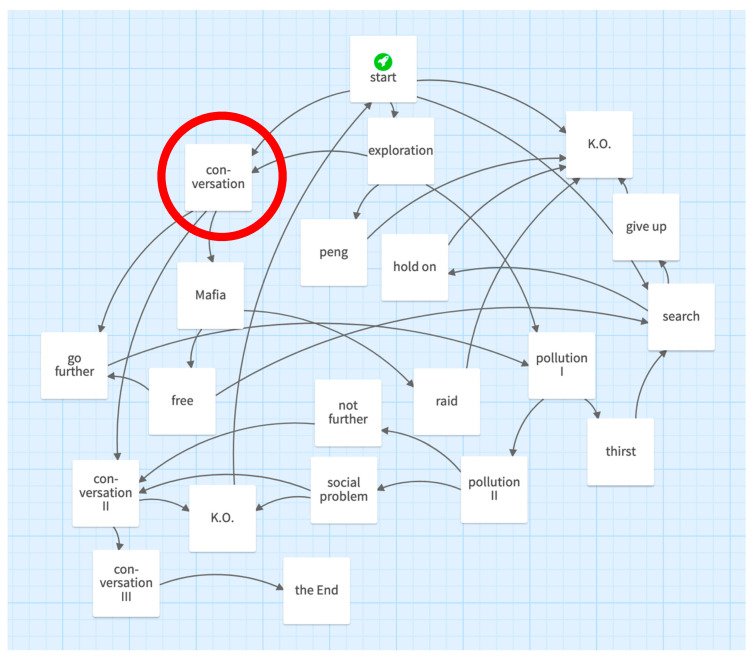
Screenshot of the Twine story “Du und Myanmar” (You and Myanmar) designed by pupils (Group F) (translated).

The narratives in Twine consist of passages that are connected with links. One finds the structure of the story “You and Myanmar” in Figure 3. In this text adventure, the player wakes up in an unknown place (start). Among other possibilities, the player has the opportunity to talk to some locals (conversation) or to explore the landscape (exploration). With a bit of luck, the player will find their way through this and other conversations with the Burmese mafia (Mafia) and other locals (conversation II) and will not be knocked out (K.O.). In doing so, they can find out information about the social conditions (social problem) and environmental pollution (pollution) in Burma. All the white squares are called passages, connections are symbolized by arrows, and the start is highlighted by a small rocket symbol. An explanation of the circled passage follows. 

The passages are the white squares in Figure 3 and are identically structured: A passage contains a title, text and links to other passages. The text of the circled passage follows as an example. Links to other passages are shown in double square brackets, as in Twine. All the text was translated by the authors.

*Conversation*
*Your conversation partner tells you in rough English how much nature suffers from tourism. For example, waste water is simply discharged into Inle Lake. According to him, however, the population benefits from tourism.*
*[[1. Interesting → go further]]*
*[[2. Ask how he benefits → Mafia]]*
*[[3. continue listening → conversation II]]*

We analyzed the text adventure games in a quantitative way. We counted all the passages (21 in Figure 3), and we divided the total number of words in the entire text adventure by the number of passages. In our example, 550/21 = 26.2. This should help to compare the group results (games) in respect of the construction. We used this method to distinguish between text adventures that were rich in passages but poor in words and those that were poor in passages but rich in words.

#### 3.3.3. Evaluation Questionnaire

In order to gather the students’ opinions about working with Twine and text adventures, we developed a short questionnaire. This information is helpful in answering the question of how to evaluate working with Twine. The questions were adapted from a previously produced questionnaire, which was applied in previously published studies for the evaluation of our university seminars [80]. The questions related to how the participants liked working with Twine, what problems they encountered and how they solved them. Only open questions were used. We used 3 deductive categories (like, challenge and solution) for analyzing the 14 questionnaires, and we followed Mayring’s [78] approach for summarizing, explicating and structuring [78] (p. 268f) the pupils’ answers. We worked with a simple spread sheet. Table 2 provides an overview of the coding manual. 

#### 3.3.4. Context Material: Plenary Discussion and Rating of the Games

The plenary discussion was recorded and transcribed with transcription software (easytranscript). We used the ratings of the games and the recorded audio group discussion as context material, explaining and interpreting unclear cases. Both data sets were useful during the analyses of the evaluation questionnaires and the construction process. For intercoder reliability, we discussed the results and unclear statements together and with colleagues at our institute. 

## 4. Results

This section is divided into three parts. Each part covers one sub-question.

### 4.1. Evaluation of Working with Twine

In the following section, we show some results from the evaluation sheets to answer the question of “how the participating pupils evaluated their experience of using Twine”. This is interesting, as Twine offers the possibility to motivate pupils with creative tasks. The pupils mentioned positive and critical aspects of using Twine in geography lessons. The positive statements emphasize the freedom they felt whilst undertaking the work and the possibility to be creative.

*S8_2: “Very good, lots of freedom, lots of fun, very creative work”.*

It is interesting that the pupil mentioned creativity, since geography and working with computers is not the first thing that comes to mind when thinking of creative work. The comments regarding user-friendliness are largely positive, too. All the groups were successful and were able to create their own games in the given time. The following statement supports this.

*S10_2: “it was very easy to understand and use”.*

This is particularly important since teachers often argue, with respect to limited time, against innovation [81] (p. 429). Challenges were seen in the work with Twine, especially in the following interesting aspects.

*S3_3: “Develop a story, add facts, keep an overview”.*

One pupil mentioned the challenge of creating a story and integrating facts. Since narrating is not a common task in geography education, this was probably a new challenge for the pupils. The same pupil found brainstorming to an appropriate way to overcome this challenge. Obtaining an overview of the task was also mentioned as a challenge in this project-orientated work. Some pupils recommended building a mind map to form such an overview. As creative writing is rarely used in science subjects at school [60] (p. 15), it therefore might be helpful to remind students of supporting methods such as mind mapping when working with Twine. In all, the students found that Twine was a welcome change from normal lessons.

*S13_6: “I think Twine is a good way to explore and work on topics in a different way”.*

After obtaining these results, which show that the students had a positive impression of Twine, we wanted to see what the students did during the lessons.

### 4.2. Activities When Constructing a Twine Game

#### 4.2.1. The Occurrence of Text Adventure Construction Activities According to Frequency

**Figure 4:**
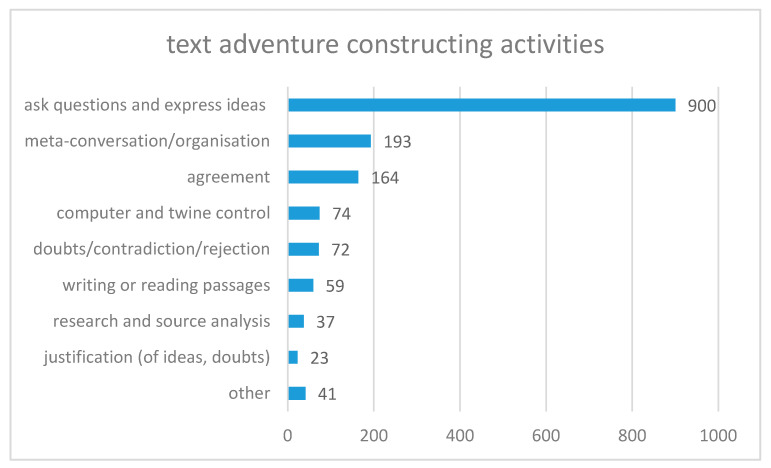
Text adventure constructing activities of the pupils. All conversations of 6 groups in two 90 min sessions are divided into text adventure construction activities (n = 1563).

**Figure 5:**
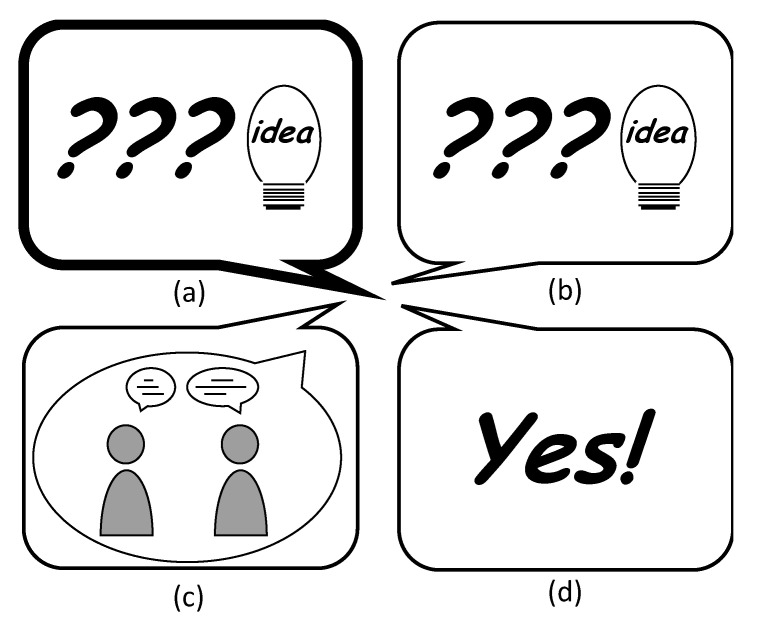
The text adventure construction activity *ask questions and express ideas* (**a**) was most often accompanied by further *questions and ideas* (**b**), second most often by *meta-conversation/organization* (**c**) and third most often by *agreements* (**d**).

**Figure 6:**
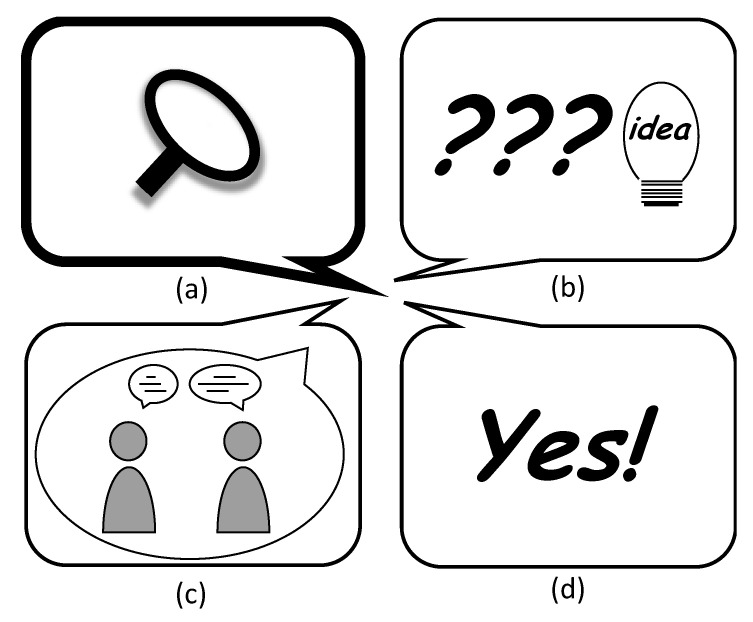
The text adventure construction activity *research and source analysis* (**a**) was most often accompanied by *questions or ideas* (**b**), second most often by *meta-conversation/organization* (**c**) and third most often by *agreements* (**d**).

**Figure 7:**
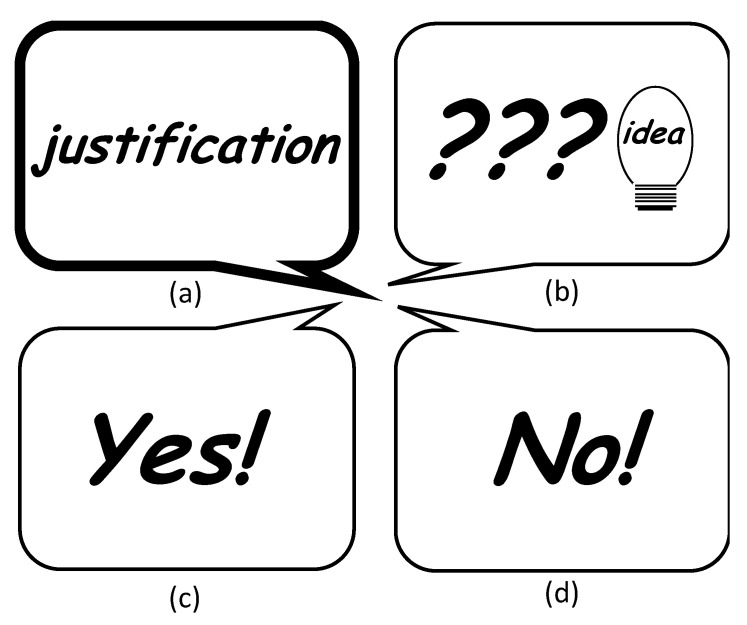
The text adventure construction activity *justification* (**a**) was most often accompanied by *questions and ideas* (**b**), second most often by *agreements* (**c**) and third most often by *doubts/contradiction/rejection* (**d**).

To answer the question of “how does the creation process work when constructing a text adventure narrative on geographical issues”, we analyzed the transcriptions from all the groups and categorized them into text adventure construction activities. Furthermore, we attempted to assign these activities to the stages of the creative collaborative model of Aragon and Williams [68]. Figure 4 shows all the activities undertaken during the construction of a Twine game, organized by their quantities. We subsequently looked more closely at which combination of text adventure construction activities occurred most frequently, which Figure 5, Figure 6 and Figure 7 illustrate.

We identified 1563 codes in all six groups of pupils in total. The majority (58%) of the codes were assigned to the text adventure construction activities of *ask questions and express ideas*. The sentence from S1 in the following conversation is an example:

*S2: locals. OK. We got it. And now?*
*S1: You’re trying to communicate with the locals?*
*S2: You just walk past the local?*
*S1: Yes. (A1_145ff)*

Figure 4 shows that constructing a game with Twine generated many new ideas, which, in our example, included the idea of communicating with a local. In the story, the local helped the player to find a way to the mainland, where the story continues. This text adventure construction activity occurred in the Create stage of the creative collaborative model of Aragon and Williams [68], with an intense interaction between the participants. As in a brainstorming process, new ideas were welcome.

About 10% of all the speech recorded was *agreements*. This suggests that, when developing games with Twine, ideas were often born in a culture of consent. An example of an agreement is found in Line 4, as S1 wants to support the idea of S2. When ideas are formulated as questions, agreements are important to express support and to steer the story. However, agreements could occur in all stages.

Approximately 12% of all the text adventure construction activities were identified as *meta-conversation or organization*. It was therefore deemed to be very important to talk about actions and to address a certain degree of organization in the group. The following lines provide an example:

*S3: But we must not waste too much time on this, because it must have something to do with tourism. (B1_108)*

In this example, S3 reminded the other group members not to spend too much time planning the beginning and formulating it perfectly. The group should stay on topic. This social process contributed to achieving a result and keeping motivation high [67] (p. 231). We can imagine that without or with less meta-conversation, a group could get lost in their own ideas. This text adventure construction activity occurred within the Frame stage, when the groups were organizing themselves [68].

About 5% of all the activities fell into the category *computer and Twine control*. This shows that the students coped well with the program. The following line is an example:

*S3: There is already a passage with this name! Now I know where the problem is. (D2_32)*

Twine, in general, is simple to use. However, there are still some problems, as in this example. Some problems occur with the linking of passages in Twine. It is very simple, but two passages or documents are not allowed to have the same name. Furthermore, not all internet browsers are compatible with Twine, but the three most commonly used browsers do work. This activity could occur in all stages of the model from Aragon and Williams.

Less than 5% of all the codes were identified as *doubts/contradiction/rejection*. That means that only a small proportion of all the ideas were rejected, and that constructing a Twine adventure game includes a lot of brainstorming with few doubts, contradictions or rejections between group members.

Less than 4% of all the activities were identified as *writing or reading passages*. This action happened when the group agreed on a formulation and tried to enter a new status quo in the construction process for the adventure game. The reading out loud of a passage helped the whole group to continue with the game construction process. In the following example, this method helped S2 to clarify the status quo of the text adventure.

*S1 (reading loud): You get out of the barrel and then you have two possibilities.*
*S2: I thought he is in a basement of a hotel. (B1_192f)*

An explanation for the high quantity of this category could be that written text is clearer and more binding than text presented orally. Furthermore, writing it down is likely to help pupils to reserve enough mental capacity to collect further ideas, as well as helping group members to see all the ideas, who may not be able to see the computer monitor because of their sitting position. This text adventure construction activity occurred mainly in the Create stage.

Only 2% of all the speech acts referred to *research and source analyses*. All the statements that referred to material analyses and research on the Internet belonged to this category. As research can be a time-consuming activity, the percentage was low but may not be too bad. It could be seen as a sign of a well-informed group in respect of previous knowledge and great understanding of the issue. Furthermore, the own imagination can be used as a source. The motivation for research could be that the pupils wanted to keep their adventure game as authentic as possible, such as in the following example. Here, the students did not know which currency was used in Myanmar and decided to research this on the Internet.

*S1: Which currency do they use? Nikki!*
*S2: Euro?*
*S1: Come on. Look it up, please! (H1_158ff)*

Another explanation is that the construction process for a creative text adventure must consider reality that is based on facts, which are identified with research. This text adventure construction activity belongs to the Focus and Create stage.

About 1% of all the speech acts were *justifications*. Justifications are clauses that provide adequate grounds or warrants for a claim [82], as in the following example.

*S3: And then at some point he has to ask, ‘why do you speak German’?*
*S2: But we can also write that he is not German, but has learned German because it is written in the text that they learn languages through tourism. (C2_145ff)*

In this example, S2 contradicts the implicit assumption that someone who speaks German must also be German, formulated by S3. Argumentation is important when constructing text adventures, as these games often rely on cause–effect relationships. However, as argumentation is important for understanding, the result seems to be disillusioning. In respect of the problem-solving process, we would expect more justification because problem-solving requires the production of arguments [83] (p. 5). A reason for this result could be that oral arguments are often without pronounced justifications. Instead of pronounced justifications, people make references to common knowledge that does not have to be pronounced [71] (p. 47). Another explanation could be that *doubts/contradiction/rejection* are often justified when creating a text adventure. However, as Twine is able to incorporate an almost unlimited number of ideas, *doubts/contradiction/rejection* were very rare (in 5% of all cases), and we therefore find minimal *justifications*. This text adventure construction activity could occur in the Focus, Frame and Create stages. After explanations of the different amounts of text adventure constructing activities, we tried to find out the combination in which these text adventure construction activities occurred most frequently.

#### 4.2.2. The Combinatorial Appearance of Text Adventure Construction Activities

In Figure 5, we focus on the text adventure constructing activity *asking questions and expressing ideas* because it was the most frequent activity, and point out three other text adventure constructing activities that occurred most frequently together with it.

Many ideas or *ideas* formulated as questions followed or were followed by other ideas or questions. A reason for this could be that good ideas need to mature, as the collaborative creative process by Aragon and Williams [68] requires in the Create stage. This suggests that good ideas come from questions and iterative improvements, which could reflect an intense brainstorming process. In the following example, S1 was formulating the status quo of the story: “you see a man in a suit waving”. We found several ideas or *ideas* formulated as questions in Lines 2, 3 and 4. All the statements were ideas for the progression of the story. In Line 5, S1 summarized the ideas (trust, distrust and running away). Answering a new idea with another idea could be an automatic contradiction or just another alternative idea, too, as it is in the following example.

*S1: Yes, you see a man in a suit waving to you.*
*S3: You go there. Or do you run away?*
*S1: And slowly you get nervous.*
*S2: What do you do?*
*S1: trust, distrust, running away.*
*S3: yes, whatever!*
*S1: OK*
*S3: I think this could take forever until we are finished. (B_211)*

Many other ideas or *ideas* formulated as questions were accompanied by *meta-conversation/organization*. In the example, represented by the last line above, S3 finished the process by an indirect reference to the limited time. This could be an indication that ideas need to be framed and placed into context, which is what Aragon and Williams [68] describe with frames. Furthermore, S3 could have also been expressing criticism of the whole design process with this statement. However, the group continued the construction process following this comment. The same motivation could be behind the combination of *ideas* or ideas formulated as questions with *agreements*, an example of which is found on Line 6. This is where S3 agreed to the proposed options. This was a verification by one group member and part of the Create stage from Aragon and Williams [68]. It was also assumed that authority structures and the group climate were influencing the situation [67] (p. 230). After the formulation of ideas, they need to be classified relating to the context, and agreements attach value to ideas. In the common brainstorming process, no agreement can be synonymous with rejection.

In Figure 6, we focus on the text adventure construction activity *research and source analysis* because gathering information and the ability to analyze it is a key competence in geography and belongs to the competence area “acquisition of knowledge”, an area of competence in German education standards for geography [51] (p. 18). Furthermore, it seems to be important in the Create stage of the collaborative creativity model from Aragon and Williams [68]. We point out three other text adventure construction activities that occurred most frequently together with it.

We categorized a large number of text adventure construction activities as *research or source analysis*, and these followed or were followed by *ideas or questions*. The pupils developed research requests from ideas, and these, in turn, emerged from them. In the following example, the idea of putting the story on a desert island already existed, and the island on which the story actually took place was researched. *Research or source analysis* and *questions or ideas* belong to the Create stage from Aragon and Williams’ model [68]. In the following example, a part of the search request (Line 1; Lanbi Kyun is a Burmese island) went on to be the title of the group’s adventure game at the end.

*S2: can you look quickly for, Lanbi Kyun or search for another lonely island?*
*S1: okay.*
*S3: How do we make the first decision now?*
*S1: Is in Myanmar.*
*S1: it’s cool.*
*S3: How do we continue now? We can’t just write now, who do you ask? Well.*
*S2: No, but where are you going, behind you is the beach, in front of you the sea and next to you the forest.*
*S1: Where are you going? Along the beach or into the forest?*
*S2: Yes! (C1_ C122ff)*

One other text adventure construction activity that occurred in combination with *research or source analysis* was *meta-conversation or organization*, and example of which is on Line 1: S2 gave the order to research Lanbi Kyun or a lonely island. Search requests were also delegated in group work. In some teams, it looked like the division of responsibilities was helpful in the Create stage for an intense thematic engagement. The delegation of work falls within the Form stage.

The text adventure construction activity *research and source analysis* was also accompanied by *agreements*, in our example. After the delegation of a search request, the *agreement* can act as confirmation of the request. In the example, found it in Line 2, S1 continued with a research order and with the evaluation “it’s cool”. This evaluation was just an interim confirmation before continuing with the task. After new ideas, another *agreement* is found in the last line of our example.

In Figure 7, we focus on the text adventure construction activity *justification* because in problem solving, the production of arguments that require justifications is essential [83] (p. 5). Therefore, in Aragon and Williams’ four-stage model, we expect *justification* mainly in the Create stage (see Figure 1). We point out three other text adventure construction activities that occurred most frequently together with it.

Communication categorized as *justification* often followed or was followed by *ideas or questions*. Some ideas were justified, and some of these justifications triggered new ideas. This fits the Create stage, when new ideas are born. All the justifications were related to the structure or logic of the story, such as in the following example. As we found just 1% *justification*, many new ideas did not need or did not get justification. A reason could be that some justifications are part of common knowledge and therefore do not have to be announced [71] (p. 47). Sometimes, a *justification* was given immediately with a new idea, as seen in the following example in Line 2: S2 argued for the beginning of their story to be at Yangon because of the possibility of arriving at the airport. Such argumentations with content references are part of the Create stage.

*S1: Where are we supposed to go?*
*S2: I thought maybe Yangon, because that’s where the airport is.*
*S3: Yes!*
*S2: The international airport. Yeah, and that’s where we arrive. Yeah, let’s just go there.*
*S1: I thought we would arrive by boat.*
*S3: Nah, just let us fly.*
*S1: So, should I write like this, you arrive in Yangon.*
*S2: You arrive there from a long flight. […]*
*S1: 16 h with one stop but. [typing]*
*S3: Easy after a long and exhausting flight. [typing]*
*S2: after a long and exhausting journey, sounds cooler.*
*[typing, clicking] D1_17ff*

*Justifications* were often accompanied by *agreements*. In the example represented in Line 3, this agreement did not come from the same pupil who asked the question (Line 1) and could also be seen as support. Of course, *doubt/objection/rejection* also occurred in combination with a *justification*. We identified doubt from S1 in Line 5 and an attempt by S3 to remove it in Line 6, where S1 asked if the story could start with an arrival by boat in Myanmar but S3 just wanted to fly. There is no justification, probably because arriving by plane is more common in this group or because the argument from S2 in Line 2 was convincing. Following this example, *doubts/objection/rejection* were considered to be part of the Create stage in the Aragon and Williams model [68], as agreements are.

### 4.3. Differences between Groups Constructing a Twine Adventure Game

To answer the question of what differences there were between the groups that used Twine to construct creative text adventures on a geographical issue, in this section, we first show the results of the game analyses and second show the group activities constructing a Twine adventure game for each group individually.

#### 4.3.1. Differences between Groups in the Constructed Text Adventure Game

**Figure 8:**
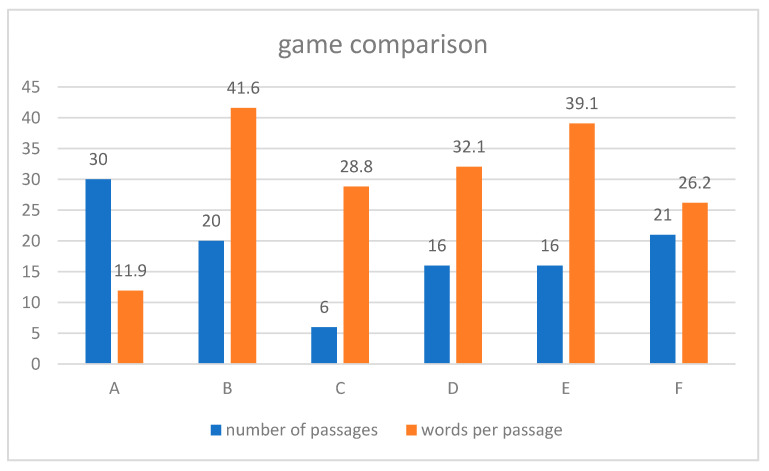
Game comparison by number of passages and words per passage.

To compare the text adventures, we counted the words and passages in all the games. We distinguish between text adventures that were rich in passages but poor in words (e.g., Group A) and those that were poor in passages but rich in words (e.g., Group B). This relationship is likely to have had an influence on the construction process, which we discuss in Section 4.3.2. Figure 8 shows the results for each group.

Group A wrote the most passages (see Figure 8). They created a kind of a labyrinth at the beginning of the story “Stranded” (originally “Gestandet”) with some dead ends. Only after several maneuvers in the wrong direction did we find the way to the main story. Thematically, this adventure game is rather flat; after the plane crashes on an island, the aim is to look for ways out. The player meets a local, and after answering a question correctly, the players get help to get to the mainland by boat. There, the player can look for a telephone, a ride to the city center or a job. The job is in agriculture, as that is where many jobs are found. That is the end of the story.

One consequence of the intense creation process for this labyrinth was that at the beginning of the story, a small number of words were used. In contrast to this, the most words per passage were written by Group B.

The adventure game from Group B “Tourism—your chance for a new life?” (originally: “Tourismus—Deine Chance auf ein neues Leben”) is written in a literary manner. A consequence of this is that this section contains the most words per passage (see Figure 8). The following passage is an example:

*Water*
*You show up with your eyes closed. The water feels warm. Where could you be? You feel something around you and open your eyes. Scared, you realize that the water surface is covered with plastic! A beach stretches out before your eyes. What area do you swim to?*
*[[1. Full beach → many tourists]]*
*[[2. Empty beach → no tourists]]*

The rhetorical question specifically (where could you be?) and the fact that they talk about the feeling (the water feels warm) suggests a literary style. In this story, the player is a blind passenger who flees from Bangladesh due to unemployment in an old cargo ship. The player can leave the boat by jumping into the water, hiding in a barrel or dressing up as a sailor. All roads lead to Myanmar, where the player sees rich tourists living in luxurious hotel resorts, and the player finds much plastic garbage on the beach. With a little luck, the player finds a job as a cleaner in a hotel. However, the money they earn is not enough to live on, so they have to decide to look for an empty hotel to reopen or to flee again. That is the end of the story.

Group C had the lowest number of passages (six; see Figure 8), because they did not save their game correctly. Their title was “Lanbi Kyun”, the name of Burmese island. Group D wrote the adventure game with the title “Trip to Yangoon” (“Reise nach Yangoon”). They let the player visit some tourist spots such as Shwedagon Pagoda, and the text transmitted some information about social problems along the way. Group E had the title “The Tourism Adventure in Myanmar” (“Tourismusabenteuer in Myanmar”). They let the player have a conversation with a local to get some information about Myanmar, the society and some environmental issues. Group F wrote a story with the title “You and Myanmar” (“Du und Myanmar”). Their story had two possible endings and was based on some luck that the player needs to “win”. There was less geographical information included.

#### 4.3.2. Differences between Groups in the Construction Process

**Figure 9:**
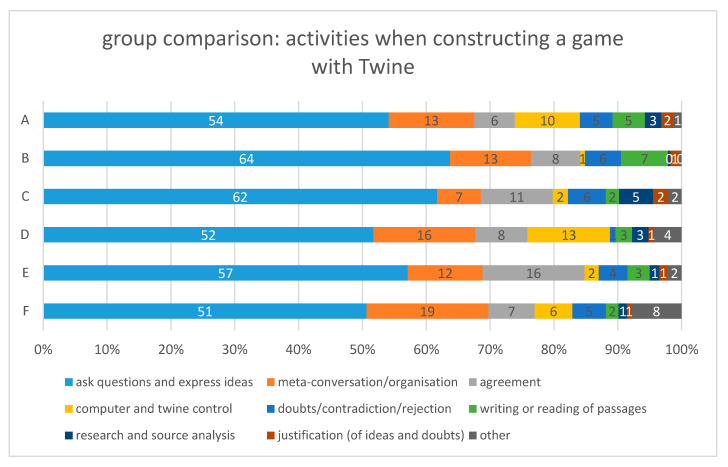
Group comparison: activities when constructing a game with Twine. All conversations of each group in two 90 min sessions are organized into text adventure construction activities (n = 1563).

The results for the group activities for each group individually are found in Figure 9. The groups are found to only differ slightly. In all the groups, the ratios between the different activities are approximately the same. This is surprising, as each group created very different adventure games to the others.

Group B had the largest amount of *questions and ideas* (64%). This could be why they created the Twine adventure game with the most words (see Figure 8). However, Group F had the lowest amount of *questions and ideas* (51%) in their conversation, but whilst they had the least words per passage, they had the highest amount of *meta-conversation and organization* (19%). This may have inhibited the creation of ideas. A reason for the high amount of *meta-conversation and organization* could be that this group only had an intense conversation about the goal of their game after about 45 min.

Group E had the largest proportion of *agreement* (16%). One reason for this could have been that the group members considered many thematic aspects in their adventure game and this provoked a lot of approval. However, Group A had the lowest amount of *agreement* but a relatively high amount of *doubts* compared to the other groups. This may be explained by the high number of passages (see Figure 8) because in Twine, it is possible to create unlimited new passages and links, so there is no need to exclude an idea or contradict a contradiction.

Group C did not save their game correctly and thus only had 2% talk about *computer and Twine control*. More computer-related (guided) conversations could have prevented this. However, Group D had the largest percentage of *computer and Twine control*. A reason could be that this group had tested its Twine adventure game intensively, and the group was therefore able to identify bugs and improve their game.

## 5. Discussion and Conclusions

With this study, we attempted to identify how pupils in small groups constructed creative text adventures on geographical issues using Twine. We first investigated how the participating pupils evaluated working with Twine. Altogether, the results suggest a positive impression gained by the students on the process, which is also supported by the fact that Twine is simple to use. Only 5% of all conversation in the groups was about *computer and Twine control*, suggesting that the groups were able to focus on content and did not have to solve many computer-related problems. The overall result was that the pupils had fun, corresponding to the results from Tan [23] (p. 220). To support storytelling, embedding facts and keeping an overview are probably some useful activities in lessons.

Secondly, we aimed to answer the question of how the creation process works when constructing a text adventure game focused on geographical issues. Altogether, writing text adventures seemed to be a very creative method, as many new *questions and ideas* were born (on average, 58% of all conversations). We discovered different text adventure construction activities that structured the group conversation, which fit into the model of Aragon and Williams [68] and explain the collaborative creativity between the Focus, Frame, Create and Complete stages. The different phases or text adventure construction activities were found to alternate in many small steps, which fits the collaborative creativity model from Aragon and William [68], because it is circular and iterative, rather than Wallas’ [65] linear model. It should be noted that the text adventure activities carried out in the study were more detailed and specific than a general model. These results thus allow a detailed structuring of the design process for text adventures, especially for the Create stage. In this respect, the model (Figure 1) can be extended (by the concretized phrases around the model) to be applicable to text adventure construction activities.

Besides the many new ideas that emerged, *meta-conversation and organization* seemed to play an important role (on average, 12% of all conversation), but it is thought that this probably depends on social and motivational team-related processes that rule group activity. This result corresponds to the results of another study on collaborative creativity [84] (p. 9). We identify only small differences in *meta communication/ organization* between the groups, which indicates that a certain amount of this text adventure construction activity is necessary [68] (p. 1880), but too much can inhibit idea generation. Doubt in group communication may lead to complex text adventures with many passages. This corresponds with previous study results that found that minority dissent supports creativity [85], but the text adventures are written in a culture of agreements (on average, 10% agreement and 5% doubt/contradiction/rejection). This fits results that most teams in general avoid conflicts [86] (pp. 142ff). Another reason for this relationship could be that Twine can incorporate almost unlimited ideas. A consequence of this capacity could be that fewer justifications are needed in the discussion phase. In this study, *justifications* were only found to occur in 1% of all conversations. This suggests that ideas do not need *justification* in a brainstorming process. Maier and Budke [71] (p. 47) also identified this deficit in investigations of the planning processes of pupils in geography lessons. It may be that justifications based on general knowledge do not always have to be given. The pupils had previously dealt with tourism in developing countries in their lessons. The curriculum lists the causes, opportunities and risks of tourism in order to understand land-use conflicts and to reflect on possibilities for sustainable tourism development. It can therefore be assumed that the pupils have a large common knowledge base on this topic, which may have led to the fact that arguments only had to be presented in group discussions when opinions or levels of knowledge differed [71] (p. 47). However, the construction of text adventures does not realize the potential of applying (geographic) situated knowledge. If we follow a constructivist learning theory, then the relation to existing (geographical) knowledge structures is fundamental to make new knowledge accessible [25,27], but the opportunity to write coherent text adventures with Twine alone does not seem to address all groups’ ability to apply (geographic) knowledge. Twine should be supported with further teaching, such as by the lesson activities undertaken in this study. The large amount of previous knowledge could also have inhibited the *research and source analysis*, which is part of the competence knowledge/methodology, i.e., one of six important competences in German educational standards in geography [87] (p. 9). This text adventure construction activity occurred, on average, in only 2% of all conversations, which suggests that the construction of text adventures is suitable for the application of existing knowledge. However, we do not know whether the construction of a text adventure also enables students to discover and explore a new subject area, as the participating students mainly used their previous knowledge.

Thirdly, we aimed to answer the question of the differences between the groups when using Twine to construct creative text adventures based on a geographical issue. Altogether, we found that the construction of a creative adventure game with geographical issues was a challenge for the groups on structural, content and cooperation levels. Teachers should support these levels through various methods, checklists or notes. On a structural level, the different passages that define a Twine game need to be connected to form a story. Writing a creative text adventure should therefore be guided by different cognitive relieving methods, which could be used in different parts of the construction process. With regard to the model of collaborative creativity [68], when groups are in the Focus stage, they need some structuring notes that would help them to reflect on the thematic complexity. When groups are in the Create stage, they may need some guidance on creative methods such as brainstorming or mind mapping. Otherwise, less-organized pupils or groups will only write simple narratives that are likely to not reflect the complexity of reality. On a content level, text adventures need to focus on one specific topic. The consideration of geographical content could possibly be supported by a checklist or a list of questions (for example, what is the main geographical issue that your text adventure covers?). At the level of cooperation, writing text adventures in small teams requires a certain amount of cooperation between team members. Since these narratives are logically structured and composed of interwoven decision-making situations, they can only be processed with divided responsibilities to a limited extent, as is the case in other group processes such as poster design. The group cooperation could be supported by organization notes that help to define different responsibilities such as researching or writing.

Overall, the groups only differed slightly in the use of text adventure construction activities. This may suggest that the application of a general model, such as that of Aragon and Williams [68], could support group processes. Teachers should take this into account in class. Thus, clarifying the possible sequence of collaborative creativity could help to reflect on group processes during the work. A comparative intervention study could provide answers to this question. However, the constructed games differ in the content, number of passages and words per passage. The results show that successful groups created stories on the basis of a journey, escape or misfortune. The successful stories used adverbs such as “rapidly” to emphasize psychological proximity in stories, and rhetorical questions (where could you be?), and they talked about feelings (the water feels warm), which suggests literary competence [35]. These strategies could be fruitful for teaching in school, such as when writing text adventures, enabling pupils to apply geographical knowledge in new contexts. Furthermore, the successful stories use more words per passage than the less-successful ones. Certainly, there is also an upper limit to the length of the passages, but we did not investigate this.

The findings of this study have some limitations. We only analyzed a small group of students, and the results therefore cannot be generalized. Further studies should consider more students. Furthermore, we limited the text adventure games to our specific topic (Tourism in Myanmar: threat or opportunity). A fully creative process should include more freedom, as we do not know how these students would create text adventures on other topics. This should be considered in further studies. The fact that the students also had some previous knowledge of the topic may have also affected their creative process. Further studies could use interviews or questionnaires to find out more about previous knowledge. This could help to enlighten us as to the low number of justifications, too. We limited the creative process to two 90 min lessons. More time flexibility could change the results. We analyzed audio data. Surely, an analysis of video data would have enriched the group process with results regarding gestures and facial expressions. In further studies, these would be explicitly considered. In addition, one of the researchers taught the two lessons and could have influenced the results (even unconsciously) during the recording. We have tried to take this into account by clearly separating the phases with group work and phases with teacher participation (e.g., plenary). Further studies may try to draw on other teachers in this respect.

The further exploration of narratives in geography education is urgently needed to illuminate the relationship between learning geographical facts and writing a structurally attractive text adventure. There is a lack of knowledge about the use of geographical narratives in geographical school lessons. An interesting future study would be to look into further strategies in writing and analyzing narratives to implement narratives in geography lessons. Furthermore, it seems to be necessary to develop a model of writing and reading geographical narratives to analyze and support pupils’ narrative skills. However, further research needs to be undertaken focused on the collaborative creative construction process in respect to the incorporation of geographical facts if we want to educate pupils on methods of applying geographical knowledge in new contexts.

## Figures and Tables

**Table 1:** Overview of the coding manual text adventure construction activities.

Category	Explanation	Example ^2^
Ask questions and express ideas	All questions and all new ideas on content ^1^.	*S1: You’re trying to communicate with the natives? (A1_146)*
Meta-conversation/organization	All phrases about the creation process or phrases about group organization.	*S3: But we must not waste too much time on this, because it must have something to do with tourism. (B1_108)*
Agreement	All agreements.	*S1: I don’t think what you are doing is stupid at all. I think it is really good! (C2_12)*
Computer and Twine control	All phrases about Twine or computer use.	*S3: There is already a passage with this name! Now I know where the problem is. (D2_32)*
Doubts/contradiction/rejection	All doubts, contradictions or rejections.	*S2: Okay, but this is also a rather unrealistic number. (E2_30)*
Writing or reading passages	All reading out of written text.	*S1: You get out of the barrel and then you have two possibilities. (writing) (B1_192)*
Research and source analysis	All phrases that ask for research or present researched results ^1^.	*S1: Which currency do they use? Nikki!* *S2: Euro?* *S1: Come on. Look it up, please! (H1_158ff)*
Justification (of ideas and doubts)	All justification of ideas or doubts/contradictions/rejections.	*S3: And then at some point he has to ask, ‘why do you speak German’?* *S2: But we can also write that he is not German, but has learned German because it is written in the text that they learn languages through tourism. (C2_145ff)*
Other	Other phrases.	*S3: I don’t care what you prefer to do. (D1_12)*

^1^ Double coding was permitted. ^2^ All examples translated.

**Table 2:** Overview of the coding manual evaluation.

Category	Explanation	Example ^1^
Like	All answers with a positive note.	*“Very good, it was fun to develop own adventutres”. (S3_2)*
Challenge	All answers that identify a challenge.	*“little time” (S6_3)*
Solution	All answers that identify a solution.	*“nothing at all; a few passages left out”. (S6_4)*

^1^ All examples translated.
